# De l'Influence des Professions sur la Durée de la Vie. Recherches Statistiques

**Published:** 1836-01

**Authors:** 


					Art. VIII.-
-De VInfluence des Professions sur la Duree de la Vie.
Re-
cherchesStatistiques. Par LeDR. H. C. Lombard,Medecindel Hopital
Civil et Militaire, de Geneve. Geneve. 4to. pp. 44. 1835.
Dr. H. C. Lombard, of Geneva, has recently examined the influence
of different professions on the duration of life; and as it is the first time
that this important question has been treated to any extent, on statistical
data, we shall present an abstract of the most interesting- results Dr.
Lombard's industry has enabled him to collect. His investigation
embraces the deaths of 8488 males, aged sixteen years and upwards,
whose ages and professions are recorded in the Mortuary Registers of
Geneva, between the years 1796 and 1830. These 8488 persons lived,
on an average, fifty-five years, which were assumed as a mean term ; and
according as the mean age at the death of those attached to any pro-
fession, exceeded or fell short of this term, the profession in question
was adjudged favourable or unfavourable to longevity.
Geneva is a small city, of 26,000 inhabitants; and the number of
deaths, in many of the professions examined by Dr. L., was too small
to furnish satisfactorily the mean age (vie moyenne) of each ; hence all
professions in which at least forty deaths were not recorded, are excluded
from the first Table.
Table i.?Presenting the Mean Age, at Death, of 6338 Males, dis-
tributed in 42 different Professions.
71
275
52
80
476
67
152
41
202
47
40
152
99
94
120
1073
124
43
176
117
267
Professions.
Arerage
Age.
Magistrates
Persons with a fixed In-
come, (Rentiers)
Protestant Clergymen
Retired Officers
Merchants
Clerks in Public Offices
Goldsmiths
Weavers
Gardeners
Founders
Criers, &c.
Different Tradesmen
Wood-cutters
Hair-dressers
Tavern-keepers
Watch-makers
Masons
Tanners
Carpenters
Spring-makers
Agriculturists
69-1
65-8
63-8
63-6
620
61-9
61-6
60-5
60-1
59-4
59-1
59-0
58-8
57-5
56-3
55-3
55-2
55-2
55-1
54-8
54-7
Number
of
Deaths.
179
63
41
376
247
97
41
77
171
48
370
125
78
46
82
143
138
46
75
62
65
Professions.
Engravers
Farriers, Blacksmiths
Printers
Shoemakers
Tailors
Coopers
Surgeons, Apothecaries
Butchers
Day-labourers
Porters
Watch-box makers
Calico-printers
Carriers
Writers, Writing-masters
Bakers
Joiners & Cabinet-makers
Jewellers
Boatmen
Enamellers
Locksmiths
Painters, Varnishers
1836.] Dr. Lombard on the Influence of Professions on Life. 195
We proceed to give Dr. Lombard's comments on these and other
numerical facts which appear to us of most importance in his memoir,
or for which the data were most adequate; reserving the few remarks
we have to offer to the end.
From this table (I,) it may be inferred that the duration of life varies
among different classes of the general population; in the first rank are
found men who, on an average, attain the seventieth year ; others do not
survive the forty-fifth year: or, to express the fact more generally,
according to the circumstances in which certain classes of the same
population are placed, their lives may be extended or abridged more
than one third. Mr. Finlaison deduced, from his calculations, that the
duration of life was the same in all classes who insure their lives, and that
in this respect, it was of little importance whether a man was robust or
delicate, provided he could save a sufficient sum to pay annually to an
insurance office. If it were necessary to combat such a paradox by
figures, the preceding table, demonstrating the variations of longevity in
different professions, would furnish a victorious reply to the arithmetician
cited. On examining the table (I,) the class of persons on whose life
property is sunk is seen not to be indifferent; in selecting a magistrate
or a clergyman, instead of a writing-master or an author, each belonging
to the class of liberal professions, the chance of obtaining a long annuity
would be greatly augmented. But it is not enough to establish on
positive documents the influence of professions on the duration of life;
we must endeavour to ascertain to what causes such a result is re-
ferrible.
The principal circumstances which Dr. L. thought worthy of investi-
gation, were : the influence of riches and of poverty ; of a liberal educa-
tion ; of certain circumstances which shorten existence by developing
phthisis; and finally, of accidents to tvhich some workmen are ex-
posed.
Influence of Riches on the Duration of Life.?In some professions, a
certain degree of affluence is enjoyed; in others, on the contrary, the
gains and salaries are so low as to keep the workmen in a constant state
of poverty ; and between these extremes there is a middle class,* neither
oppressed by poverty, nor saturated with riches. Hence all professions t
may be divided into three classes : the rich and liberal; the middle; and
the poor, comprehending labourers, workmen, and the most abject opera-
tives. The application of this classification is necessarily somewhat
arbitrary, but Dr. L.'s practical acquaintance with the Genevese popu-
lation enabled him to obviate the difficulties left by theory; for example,
he placed some professions, such as drapers, money brokers, &c., appa-
rently belonging to the middle class, in the first; as these persons in
Geneva are ordinarily wealthy.
The mean age at death (vie moyenne,) of each of these classes, also
the mean age in each class of those below, and of those professions in
which the mean age surpassed fifty-five years, was as follows.
* These classes, in the small republic of Geneva, do not, as the reader will conjec-
ture, exactly correspond to the three classes into which English society is ordinarily
divided.
O 2
196 Dr. Lombard on the Influence of Professions on Life. [Jan.
Table ii.?-Professions classed according to their Riches.
Mean Age
FIRST CLASS. at Death.
Liberal Professions; ? Men in easy circumstances - 61-0
Having a mean age above 55 ;?Magistrates, Physicians, &c. - 62-2
Having a mean age below 55 ;?Surgeons, Literary men, &c. &c. 52'6
SECOND CLASS.
The Middle Classes;?Tradesmen - - 56-4
Mean age above 55;?Watchmakers, Grocers, Carpenters, &c. &c. 60*7
Mean age below 55 ;?Tailors, Bakers, Coopers, &c. &c. - 50-5
THIRD CLASS.
The Poor;?Labourers, Workmen - - 53-6
Mean age above 55;?Wood-cutters, &c.; Grooms, Scavengers, &c. 57'8
Mean age below 55;?Carriers, &c.; Male Servants, &c. - 49*6
A comparison of the three classes in this table establishes one common
fact, namely, the influence of riches and knowledge in prolonging life;
the rich, on an average, live seven years and a half longer than the poor;
so that the station in which a man happens to be born in Geneva may
shorten or prolong his life nearly eight years. The difference between the
upper and the middle classes is 4*6 years, and between the middle and
the lower classes only 2*8 years.
It may then be admitted, that affluence exercises considerable influence
on the duration of life. Some years ago, Dr. Villerme arrived at the
same conclusion, by an entirely different method, applied to another
population ; he shewed, that in the several quarters of Paris, the mor-
tality augmented in proportion to the poverty and wretchedness,?that
as these prevailed life was shortened.
On enquiring how affluence prolongs life, we discover two kinds of
influences, which react on each other. The first, entirely physical, is
the diminution of bodily suffering by a sufficient supply of food, and by
the protection afforded against all atmospheric severities ; the other, de-
rived from a liberal education, excludes gross excesses, induces men to
lead a life more conformable to the laws of Hygiene, and more especially
to take proper care of themselves in derangements of health.
Mr. Madden, an English writer, from a comparison of the ages learned
men attained, has inferred that the study pf the arts and sciences is
remarkably favourable to longevity; he found, from a certain number of
cases, that natural historians lived, on an average, 75 years; philoso-
phers, 70 years; painters and sculptors, 70 years; lawyers, 69 years;
physicians, 68 years; divines, 67 years; philologists, 66 years; musi-
cians, 64 years; novel writers, 62| years; dramatists, 62; writers on
natural religion, 62; and poets only 57 years. It is probable that
this scale of longevity is considerably affected by the degree of diffi-
culty in obtaining a reputation sufficient to carry a writer down to pos-
terity in the several departments of science, art, and literature. A
naturalist, a philosopher, or a lawyer, cannot so easily win celebrity as
a novel writer, a musician, or a poet.
1836.] Dr. Lombard on the Influence of Professions on Life. 197
In the professions that follow, shewing the influence of deleterious
vapours, of dust in the workshops, and of accidents to which artisans are
exposed, some are included in which the number of deaths did not exceed
seven or eight; in these cases greater attention must be paid to the mean
of the class than to each individual group.
Table hi.?Shewing the Mean Longevity in Professions exposed to
the Action of Vapours.
Professions. Mean Age.
Hatters - - 50-9
Gilders - - 51*7
Painters in design - - 57'5
Enamellers - - 48*7
Founders - - 59*4
Professions. Mean Age.
Varnishers - - 44*3
Tinmen - - 45*6
Locksmiths - - 47*2
Blacksmiths - - 545
Mean age at death - 51*1
Mean age of their Class 56-0
From this table it appears, that workmen exposed to the inhalation of
mineral and vegetable vapours, only attain a mean age of fifty-one years,
five years under the mean age (fifty-six,) of the middle class, to which
they appertain.
The professions in the next table respire an atmosphere more or less
impregnated with suspended particles of a mineral, vegetable, or animal
nature.
Table iv.?Professions exposed to the Action of Particles suspended
in the Atmosphere; namely, to
1. Mineral Particles.
Mean Age.
Pavers - 58*2
Dustmen - 56*0
Cutlers - 57*0
Pinmakers - 65'4
Mean Age.|
Polishers - 53*7
Sculptors - 363
Stonecutters - 34*4
Masons - 55-2
Mean Age.
Plasterers - 45*5
Terrace-makers - 58*0
Mean age at death 52 0
2. Vegetable Particles.
Millers - 420
Bakers - 49*8
Hair-dressers - 57'5
Coalmen - 55*1
Coal-measurers - 59-1
Chimney-sweepers 45-0
Mean age at death 51*4
3. Animal Particles.
Hatters - 50-9
Upholsterers - 53*0
Skinners - 70*0
Brushmakers - 50*1
Harness-makers - 60-4
Mattress-makers - 60-3
Mean age at death 57'5
The mean age of workmen respiring in a dusty atmosphere is fifty-three
years and a half; it is longer in men exposed to animal particles in
suspension than in the rest of the group ; a result different from that
obtained in Dr. L.'s researches on phthisis, which is most frequent in
workmen inhaling animal and mineral particles. When the foreign
bodies in the atmosphere are in the state of vapour, they are more
readily absorbed by the mucous membrane of the lungs, and appear to
198 Dr. Lombard on the Influence of Professions on Life. [Jan.
act more fatally on the economy than the finest powders; the relation of
the two deleterious causes is as 53*5 to 51.
Influence of a Sedentary Life.?Workmen who lead an active life, and
regularly exercise their muscular powers, are placed in hygienic circum-
stances very different from those who are habitually shut up in work-
rooms or factories, where their muscles remain comparatively inactive.
To determine the influence of exercise on longevity, we are compelled to
pass over the third class of labourers who, by a sort of compensation,
pursue their unthrifty occupations in the open air: the first two classes,
separated into sedentary and active professions, alone present comparable
results.
Table v.?Influence of a Sedentary and an Active Life on Longevity.
FIRST CLASS.
Liberal Professions;?Men in easy circumstances.
1. Sedentary Professions. Mean Age.
Druggists, Clerks, Schoolmasters, Solicitors, Literary Men, &c. 58*5
2. Active Professions.
State Functionaries, Physicians, Surgeons, and Brokers - 60'1
SECOND CLASS.
The Middle Classes;?Tradespeople.
1. Sedentary Professions.
Confectioners, Watchmakers, Engravers, Sculptors, &c. - 55*1
2. Active Professions.
Carpenters, Gardeners, Butchers, Bakers, &c. - - 56*3
A comparison of both classes leads to the conclusion, that existence is
shortened by a sedentary life; in the first case 1*6, in the second 1*2;?
nearly a year and a half. A sedentary life is injurious then ; but only
to a very limited extent.
Accidents and Suicide.?Circumstances by which men are exposed to
fatal accidents, or prompted to self-destruction, are so inherent in some
professions, as to merit consideration under this head.
Table vi.?Frequency of Suicides in different Professions.
Suicides.
First Class j?Persons in easy circumstances - 10
Second Class;?Tradespeople ... 40
Third Class j?Artisans ana Labourers - 7
57
Proportion of Suicides.
First Class 1 in 33 }
Second Class 1 in 25 > 1 Suicide in 27'8 Deaths.
Third Class 1 in 39 5
The proportion of suicides is greatest in the middle class; least among
the poor. So far as Dr. L.'s researches extend, it may be inferred that
in Geneva it is not the poor, but the middle classes, who are most
exposed to vicissitudes of fortune, that, when their resources are unex-
pectedly cut off, resort in despair to suicide.
1836.] Dr. Lombard on the Influence of Professions on Life. 199
With regard to deaths from violence, or fatal accidents, 352 in 8488
deaths are recorded (1 in 24), but this must not be taken for the mean
of Geneva, as the table includes a period of civil convulsion. If the
accidental deaths are deducted, the mean age of the rest at death will
be 55-9 years; consequently, the mean age of the entire population is
not abridged quite one year by accidents. In some professions, never-
theless, life is considerably curtailed by violent deaths; for instance,
coachmen and carriers, if accidents were deducted, would live to a mean
age of 56-5 instead of 48*2 years.
Table vii.? The Proportion of Deaths from Accidents, in eight
Professions.
Professions.
Violent
deaths.
Total
deaths.
Proportion
of
violent deaths.
Vie
moyenne.
Vie moyenne,
afterdeductiug
violeut deaths.
Butchers
Boatmen
Carpenters
Slaters
Coachmen
Tinmen
Masons
Emmenageurs
(House-furnishers, Bro-
kers, or Upholsterers)
3
6
12
7
7
4
12
7
58
77
46
176
26
90
39
124
52
in 25
8
15
4
13
10
10
7
53-0
49-2
551
47-7
48-2
45-6
55-2
60-0
53-1
51-3
55-7
48-8
56-3
47-0
55-6
59-1
630 1 in 11
51-4 53-7
These classes of men would have lived, on an average, two years
longer but for accidents, by which one in eleven of them perished.
In fine, the influences of professions may be arranged in two classes.
1st. Those favourable to life :
Affluence ... 7*5 years.
Exercise - - - 1-4
2d. Those unfavourable to life'.
Poverty - 7*5
Mineral and Vegetable Vapours - 4-9
Particles suspended in the Atmosphere 2 5
Total Accidents - - 2-3
Sedentary Life - - 1*4
In the above abstract, we have endeavoured to condense the results of
Dr. Lombard's extensive, novel, and valuable investigation ; but we
cannot entirely admit, with that ingenious statistician, either the facility
of recognizing the influence of professions on the general mortality, or
that the extent of that influence has yet been indubitably discovered.
For the mortality of a profession, or of any class, is only accurately
expressed by the relation of the number dying to the living at all ages
in a given time: and no attempt has hitherto been made in Geneva, or
elsewhere, to determine the numbers or the ages of the living and the dying
in any one profession; indeed such an undertaking, on a comprehensive
scale, would be attended with the utmost difficulty, as was experienced
in the last census, when parliament only attempted to ascertain the
total number of persons attached to each profession in this country. No
200 Dr. Lombard on the Influence of Professions on Life. [Jan.
doubt the mean age at death of persons in different professions, deserves,
on this account, our serious attention; it furnishes an approximation to the
truth; and where the professions compared are stationary, or are annually
recruited at the same rate, the individuals of 20, 30, 50, 60, &c. years
of age remaining nearly in the same proportion, the result would not.
deviate much from that furnished by the other method. But if from any
cause a particular profession is flourishing and increasing,* the number
of young men will be disproportionably greater than that of old men
living, and looking only at the mortuary register, or only taking the
mean of the ages at death, the longevity would be apparently diminished,
not by the unhealthiness of the profession, but by the surplus of young
men numbered among its members. If the mean age at death of general
medical practitioners were now collected from the parish registers of
England, their longevity would appear much below the average, because
their numbers are rapidly increasing. If the same state of things pre-
vails in Germany,?if the medical profession has been increasing in that
country,?and we believe that it has been increasing,?while on the con-
trary divinity and its professors, poorly paid, and settled in their districts
by the government, have remained stationary, the melancholy aspect
presented to both professions, by the result of professor Casper's re-
searches, will lose much of its terror. Professor Casper found that the
clergy were long livers compared with medical men; for out of 100
deaths, only forty medical men had lived to the sixty-second year, while
sixty-five divines had survived that year.
From 624 deaths in town and country, professor Casper,f of Berlin, has
deduced the following ages at which 1000 medical men, and the same
number of divines, die.
Between Ages Medical Men. Divines.
23 ? 32 - 82 - 43
33 ? 42 ~ 149 - 58
43?52 - 160 - 64
53 ? 62 - 210 - 180
63 ? 72 ?? 228 - 328
73 ? 82 - 141 - 255
83 ? 92 - 30 - 70
The mean age ^vie moyenne) of a profession of the common standard
should be exactly equal to the expectation of life at the age of enter-
ing, + the average age of entering itself. The expectation of life at 15
is 42 years,| and 42 + 15 = 57, would be the mean age of persons
entering on professions at that age: but a man does not become a
clergyman at 15; and if we admit that clergymen take orders at 25, to
enjoy the very samedegree of life their vie moyenne should be 25 + 35=60;
magistrates may enter on their functions at 40, and their longevity would
be extended to 64, because at 40 they would have escaped all the chances
of dying between 15 and 40. Dr. Lombard judiciously cautions his
* The fluctuations in some professions are very great. In the most flourishing period
of its trade, Geneva contained 700 master watchmakers, and about 6,000 workmen. At
the present time there are only 2,800 persons engaged in this business.
f Annales d'Hygiene, torn. xi.
+ According to Mr. Edmonds's mean table; where the expectation is intermediate
between the Carlisle and Northampton tables. The Carlisle table applies to the best,
the Northampton to the worst class of life.
5
1836.] Dr. Lombard on the Influence of Professions on Life. 201
readers against comparing the vie moyenne of ecclesiastics and magis-
trates with other professions, because their offices are only filled by men
of a " certain age." For the same cause we should be disposed to caution
in comparing the mean age of the poor with that of the rich; as many
remain in the inferior ranks when young, and only become affluent, or
enter on professions requiring a large capital, when they also have at-
tained a " certain age." Dr. Lombard may have obviated these objec-
tions in great part, and have avoided the inaccuracies arising out of the
irregular periods at which professions are commenced, by not descending
lower than thirty years; for at this age the majority of professions are
commenced: we are aware this would have considerably diminished the
already limited number of his observations, and must profess our thanks
for the efforts he has already made to solve a difficult but most important
problem in Hygiene. His researches extend over a long period of time,
and some of the sources of error being in opposite directions, correct
each other, particularly where several professions are grouped together.
Before adverting to Dr. Du Bois's researches on the longevity of the
medical profession, we shall adduce the few observations made by Dr.
Lombard, as they refer to the mortality of all classes of practitioners ; a
subject at present of more than usual importance, since the establishment
of a General Medical Benevolent and Insurance Society has been
agitated at the last meeting of the Provincial Medical Association.
No. of Deaths. Vie moyenne.
18 Physicians - - 66*4
41 Surgeons and Officiers de Sante - 54-0
Here is a difference of twelve years in the vie moyenne of both classes;
in part attributable to the different ages at which the officiers de sante
and physicians, commence their career : for admit that the general prac-
titioners are qualified at twenty-three years of age, physicians at thirty;
then we have 23 + (expectation of life) 39 = 62 for the mean age at
death of general practitioners; while the mean age (vie moyenne) of
physicians, found in the same way, would be sixty-four. But after this
correction, the mean age to which physicians live is sixty-six, or two
years above the standard; while the general practitioner's life (fifty-four
years,) on the contrary, falls nearly eight years below the standard: a
striking fact, which, if Dr. Lombard's observations were more numerous,
could only be attributed to a recent increase in the class of general prac-
titioners, or to the unfavourable and destructive agencies by which, in
the discharge of their painful duties, they are assailed.
According to professor Casper's observations, previously referred to,
the numbers in 100 deaths which were seventy years of age and upwards,
differed very considerably in different professions; in the following table
the results he obtained are compared with those of Dr. Lombard; the
medical profession, according to both, stands low in the scale.
Numbers who surpassed Seventy Years of Age in 100 Deaths.
Casper.
Clergymen - 42
Agriculturists ... 40
Clerks of Public Offices 33
Barristers - - 29
Schoolmasters - 27
Medical Men 24
Lombard.
46
27
36
42
33
202 Dr. Lombard on the Influence of Professions on Life. [Jan.
To determine whether literary or scientific pursuits, and that energy
of. mind which enables men to transmit their name and their works to
posterity,?whether the literarum disciplina, in the words of Celsus, ut
animo proScipue omnium necessaria, sic corpori inimica est,?is a'problem
of no inconsiderable interest. With regard to the distinguished authors
and cultivators of medical science, the following tabular view, compiled
by M. Du Bois, of the ages attained by 850 medical men who are men-
tioned in the Dictionnaire Historique de la Medecine, par Eloy, 4to.,
4 vol. Mons, 1778, offers interesting data for calculation.
? Table viii.
20
21
22
23
24
25
26
27
28
29
Total
57
83
136
202
28
18
20
27
12
37
26
14
16
15
213
116
31
100
101
102
103
104
105
106
M. Du Bois adduces this table, where rather more than forty-two in
a hundred lived to seventy years and upwards, to controvert Casper's
announcement; but it furnishes data for determining the relative longevity
of medical writers by a better method than that employed by Casper,
and for this reason we have deduced from it the expectation of life at the
age of forty, fifty, &c. years, when all, likely to obtain a place in bio-
graphical dictionaries, have established their reputation.
Table ix.?Expectation of Life deduced from the Deaths of 778
celebrated Medical Men;?compared with the Carlisle, Northampton,
and Stockholm Tables.
Ages'
40
50
60
70
Viemoyenne 40
Medical
IV]en.
2714
19-96
13-74
904
67*14
Carlisle Northampton
Table. I Table.
27-61
21-11
14-34
9-18
67-61
22.12
1700
11-92
7-08
62-12
Stockholm
Table.
18-12
13-81
9-52
5-36
58-12
The medical writers here appear to enjoy a vitality nearly as high as
that on which the Carlisle table was founded ; far surpassing the other
tables applying to populations in cities, and in unfavourable circum-
stances.
Calculated only on the deaths, these results are open to some of the
objections previously stated; but they agree very remarkably with the
1836.] Introductory Lectures?Medical Schools. * 2.03
Carlisle table throughout, and on this account merit considerable confi-
dence. Those only accustomed to contemplate fragments of life,?the
uncertain existence of individuals,?would scarcely believe, perhaps,
that the number of years 778 distinguished men have to live, at the age
of forty, may be predicted precisely, without the chance of erring more
than a few months ; yet by going through the necessary calculation^ with
the data furnished by M. Du Bois, and comparing the result with the
Carlisle table, they may convince themselves of this truth ; and "discover
that the duration of life is submitted to laws as fixed as any other natural
phenomena.

				

